# A single faecal bile acid stool test demonstrates potential efficacy in replacing SeHCAT testing for bile acid diarrhoea in selected patients

**DOI:** 10.1038/s41598-022-12003-z

**Published:** 2022-05-18

**Authors:** Aditi Kumar, Hafid O. Al-Hassi, Manushri Jain, Oliver Phipps, Clare Ford, Rousseau Gama, Helen Steed, Jeffrey Butterworth, John McLaughlin, Niall Galbraith, Matthew J. Brookes, Lauren E. Hughes

**Affiliations:** 1 The Royal Wolverhampton NHS Trust, Wolverhampton Road, Wolverhampton, WV10 0QP UK; 2 University of Wolverhampton, Wolverhampton, UK; 3 Clinical Chemistry, Black Country Pathology Services, The Royal Wolverhampton NHS Trust, Wolverhampton, UK; 4 School of Medicine and Clinical Practice, Faculty of Sciences and Engineering, University of Wolverhampton, Wolverhampton, UK; 5 Shrewsbury and Telford Hospital NHS Trust, Shrewsbury, UK; 6 Division of Diabetes, Endocrinology and Gastroenterology, Faculty of Biology Medicine and Health, The University of Manchester, Manchester Academic Health Science Centre, Manchester, UK; 7 Department of Gastroenterology, Salford Royal Foundation Trust, Stott Lane, Salford, UK

**Keywords:** Diarrhoea, Crohn's disease

## Abstract

This study examines the validity of measuring faecal bile acids (FBA) in a single stool sample as a diagnostic tool for bile acid diarrhoea (BAD) by direct comparison to the ^75^selenium-homotaurocholic acid (SeHCAT) scan. A prospective observational study was undertaken. Patients with chronic diarrhoea (> 6 weeks) being investigated for potential BAD with SeHCAT scan provided stool samples for measurement of FBA, using an enzyme-linked immunosorbent assay. Patients were characterised into four groups: SeHCAT negative control group, post-cholecystectomy, idiopathic BAD and post-operative terminal ileal resected Crohn’s disease. Stool samples were collected at baseline and 8-weeks post treatment to determine whether FBA measurement could be used to monitor therapeutic response. 113 patients had a stool sample to directly compare with their SeHCAT result. FBA concentrations (μmol/g) and interquartile ranges in patients in the control group (2.8; 1.6–4.2), BAD (3.6; 1.9–7.2) and post-cholecystectomy cohort 3.8 (2.3–6.8) were similar, but all were significantly lower (p < 0.001) compared to the Crohn’s disease cohort (11.8; 10.1–16.2). FBA concentrations in patients with SeHCAT retention of < 15% (4.95; 2.6–10.5) and < 5% (9.9; 4.8–15.4) were significantly higher than those with a SeHCAT retention > 15% (2.6; 1.6–4.2); (p < 0.001 and p < 0.0001, respectively). The sensitivity and specificity using FBA cut-off of 1.6 μmol/g (using ≤ 15% SeHCAT retention as diagnostic of BAD) were 90% and 25% respectively. A single random stool sample may have potential use in diagnosing severe BAD or BAD in Crohn’s patients. Larger studies are now needed to confirm the potential efficacy of this test to accurately diagnose BAD in the absence of SeHCAT testing.

## Introduction

Chronic diarrhoea is one of the most common reasons for gastroenterology clinic referrals^1^. Using the definition of excessive stool frequency, the prevalence of chronic diarrhoea in the western population is 4–5%^1^. One of the most common causes is irritable bowel syndrome, which is estimated to affect 11% of the global population^2^. However, approximately 15–50% of patients diagnosed with irritable bowel syndrome have bile acid diarrhoea^3^. Unfortunately, there is poor recognition of this diagnosis by professionals, with diagnostic delay often exceeding 5 years and a large unmet need in symptom control, despite treatment being available^4^.

The British Society of Gastroenterology (BSG) recommend investigating for bile acid diarrhoea in patients who have persistent diarrhoea despite normal first-line investigations such as blood tests for anaemia and coeliac serology, and stool tests for inflammation^1^. Despite this, the diagnosis of bile acid diarrhoea is often missed due to difficulties in accessing a suitable diagnostic test. As a result, patients are mis-diagnosed with irritable bowel syndrome. Currently there are three types of diagnostic tests available for bile acid diarrhoea: the ^75^selenium-homotaurocholic acid test (^75^SeHCAT), serum biomarkers of hepatic BA synthesis [7-alpha-hydrooxy-4-cholesten-3-one (C4) and fibroblast growth factor 19 (FGF-19)] and 48-h faecal bile acids. When using a diagnostic cut-off value of < 10% to correctly identify patients with bile acid diarrhoea, the ^75^SeHCAT has the highest average diagnostic yield of 30.8%, followed by the 48-h faecal bile acid test (25.5%), FGF19 (24.8%) and C4 (17.1%)^5^. However, there are limitations with each of these diagnostic methods. Specifically, the ^75^SeHCAT scan is not widely available, is time-consuming and has radiation exposure^6^; the serum biomarkers show significant variation due to diurnal variation and there is lack of availability of tests^7^; and the faecal bile acid test requires laborious organisation with patients needing to adhere to a high-fat diet, stool to be collected for 48 h and the test not being readily available in most laboratories^8^. In the UK, the National Institute of Clinical Excellence (NICE) has advised that further research is needed to establish the validity and accuracy of the ^75^SeHCAT test and other potential alternative diagnostic tests for measuring bile acid diarrhoea in people with chronic diarrhoea^9^. As a result, many physicians will opt for a trial of bile acid sequestrants with symptom improvement regarded as a positive diagnosis. This method, however, is not recommended by the BSG due to issues with patient compliance^1^. Treatment efficacy is dose sensitive as the medication is not well tolerated and achieving adherence can be challenging. Furthermore, treatment response may be open to assessment bias due to the inability to blind patients and clinicians^10^. Thus, a method for monitoring compliance would be beneficial.

Immunodiagnostik have developed an enzymatic spectrophotometric kit, marketed as a test to support diagnosis of bile acid diarrhoea through measurement of total faecal bile acids on a single stool sample. The primary aim of this study is to determine the efficacy of this kit for measuring faecal bile acids as a diagnostic tool for bile acid diarrhoea with direct comparison to the SeHCAT scan, the current gold standard diagnostic test. A further secondary aim was to evaluate the efficacy of FBA testing in assessing the therapeutic response to treatment with a bile acid sequestrant.

## Methods

### Ethical approval and good clinical practice

The study was performed in accordance with the recommendations guiding physicians in biomedical research involving human subjects, adopted by the 18th World Medical Assembly, Helsinki, Finland 1964, amended at Edinburgh in 2000. The study was conducted in accordance with the International Conference on Harmonisation Good Clinical Practice guidelines. Patient information was anonymised and any collection of patient data was in compliance of the Data Protection Act 1998. The study underwent full ethical approval by London-Stanmore Research Ethics Committee. REC ref: 16/LO/1325. Written and informed consent was obtained from all participants in the trial. All authors had access to the study data and reviewed and approved the final manuscript.

### Study design

Patients were recruited from three centres in the West Midlands, UK, that perform SeHCAT testing. Patients were recruited, and baseline stool samples collected, if they had a SeHCAT scan requested by their gastroenterologist for symptoms of ongoing diarrhoea. Diarrhoea was defined as the persistent alteration from the patient’s norm with stool consistency between types 5 and 7 on the Bristol stool chart and increased frequency greater than 4 weeks’ duration^1^. Patients were categorised into four groups: Idiopathic bile acid diarrhoea (SeHCAT positive), post-operative terminal ileal resected Crohn’s disease, post-cholecystectomy and SeHCAT negative control group. The patients with no underlying co-morbidity were contacted for study participation as soon as their SeHCAT scan was requested and depending on the result of the scan, they were categorised into either the bile acid diarrhoea or control group. The Crohn’s patients were contacted for study participation 4–6 weeks post-operatively to allow symptoms of BAD to develop. If they had symptoms of diarrhoea, a SeHCAT scan was requested. If they did not have symptoms, then a baseline stool sample was collected but a scan was not requested. The post-cholecystectomy patients were recruited as soon as their SeHCAT scan was requested, however the duration from surgery to test date varied between months to years and was dependent on when the patient presented clinically with symptoms. As per NICE guidance, a SeHCAT result of < 5% was considered severe bile acid diarrhoea, 5–10% as moderate, 10–15% as mild and > 15% as a negative result^9^. An early morning stool collection was advised, however depending on patient’s time and ability, a random stool sample was collected from any point in the day before treatment for those with a positive SeHCAT.

Patients with a positive SeHCAT result received a therapeutic trial of bile acid sequestrants; Colesevelam 625 mg once or twice daily was the first-line BAS given to patients. Patients were reviewed in a research clinic 4- and 8-weeks after treatment commencement and assessment of response was made at each review. They were required to complete a 7-day stool chart prior to their appointment where daily stool frequency and consistency (Bristol Stool Form Scale) were documented. Stool samples were also collected at the clinic appointment. Clinical response was defined as patients who had improved bowel frequency by > 50% from their initial assessment or less than 3 bowel movements per day. If patients had a partial response (< 50% improvement from their initial assessment or > 3 bowel movements/day), their colesevelam dose was increased at their first clinic appointment and reviewed in 4 weeks’ time. Any side effects of the treatment were documented, as well as review of their medication history. If patients could not tolerate the medication or no benefit was observed, they were subsequently withdrawn from the study, however their stool samples collected up to that point were still used for analysis. The study was complete after 8 weeks of treatment and patients were referred back to their original clinician. Faecal bile acid concentrations in patients before and after treatment with bile acid sequestrants were used to assess whether the Immunodiagnostik kit could be used for monitoring response to treatment.

### Eligibility

Any patient over the age of 18 years who underwent a SeHCAT scan, or had a terminal ileum resection, with symptoms of diarrhoea, and who were able to provide informed consent, were included in the study. Patients were excluded from the study if they were: pregnant or breast feeding; unable to provide written consent; known established bile acid diarrhoea; currently or previously treated with bile acid sequestrants; or recipients of antibiotics within 4 weeks of the initial trial participation.

### Protocol

Samples were analysed using the commercially available Immunodiagnostik (IDK) Faecal Bile Acids photometric kit (Ref: K7878W), provided by the BioHit Healthcare, UK. Total faecal bile acids are first extracted from the stool sample using the extraction buffer provided in the kit. The total faecal bile acid content is then measured through monitoring Thio-Nicotinamide-Adenine Dinucleotide (thio-NADH) production, by change in absorbance at 405 nm over time, during conversion of faecal bile acids to 3-keto steroids by 3-alpha-hydroxysteroid dehydrogenase. Baseline faecal bile acid results were compared to SeHCAT results. The assay was performed as per the kit insert, however an additional dilution step (1:3) was added to increase the measuring range. The kit was fully validated prior to use. The faecal bile acid assay had a limit of detection of 0.05 μmol/g and inter-assay coefficient of variation of 11.7%.

### Statistical analysis

Data were non-parametric as defined by the Shapiro–Wilk test and therefore expressed as median with interquartile ranges (IQR). Spearman rank correlation was used to measure the degree of association between SeHCAT retention times and FBA concentrations. Kruskal–Wallis test was used to assess differences between three or more groups. To assess differences between two groups, Mann–Whitney *U* test was used for unpaired data and Wilcoxon signed-rank test for paired data. For paired data comparing more than two groups, the Friedman test was used. For all statistical analyses, a p-value < 0.05 was considered statistically significant.

Receiver operator characteristic (ROC) curves were generated using Analyse-It by using SeHCAT retention of ≤ 15% as diagnostic of bile acid diarrhoea and > 15% as negative for bile acid diarrhoea, and using ≤ 10% and ≤ 5% as diagnostic of moderate and severe bile acid diarrhoea, respectively.

A priori statistical power is provided for inferential tests (denoted by the symbol 1-β), accounting for actual sample size in each case, alpha at 0.05 and based on both an average effect size (Cohen’s d = 0.5, Cohen’s f = 0.25, correlation coefficient of 0.3 respectively) and small effect size (Cohen’s d = 0.2, Cohen’s f = 0.1, correlation coefficient of 0.2 respectively).

## Results

**Figure 1:**
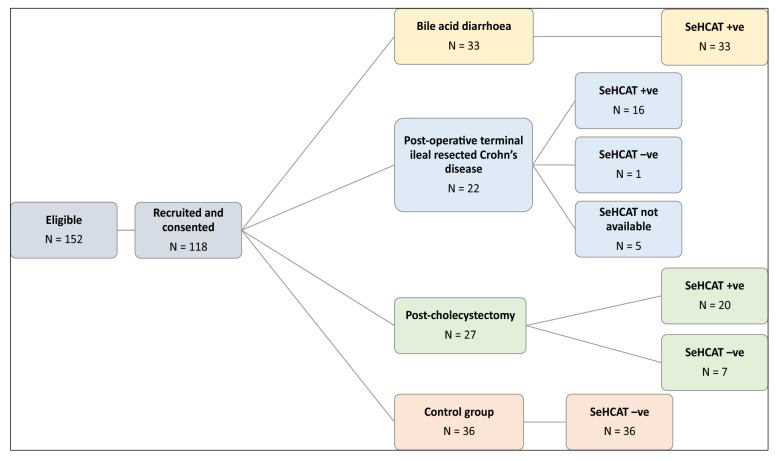
Patient recruitment into four arms of the study. 152 patients were found to be eligible of which 118 consented to partake in study. Following their SeHCAT results and based on their underlying pathology, they were categorised into either idiopathic bile acid diarrhoea, post-terminal ileal resection secondary to Crohn’s disease, post-cholecystectomy group, or SeHCAT-negative Control group.

A total of 118 patients were recruited; of these 33 had idiopathic bile acid diarrhoea, 22 were post-operative Crohn’s disease, 27 were post-cholecystectomy and 36 were from the control group (see Fig. 1 for recruitment breakdown). Of those recruited, 113 patients had a SeHCAT result available at time of analysis. The remaining five patients were recruited for the study and donated their stool sample but then did not attend for their SeHCAT test. As our study directly compares faecal bile acid concentrations with SeHCAT results, these five patients were subsequently excluded from the analyses. The mean age was 49.4 years of age and 60.2% of patients recruited were female. The full breakdown of patient demographics can be found in Supplementary Table 1.

### Faecal bile acid results in correlation with SeHCAT levels

**Figure 2:**
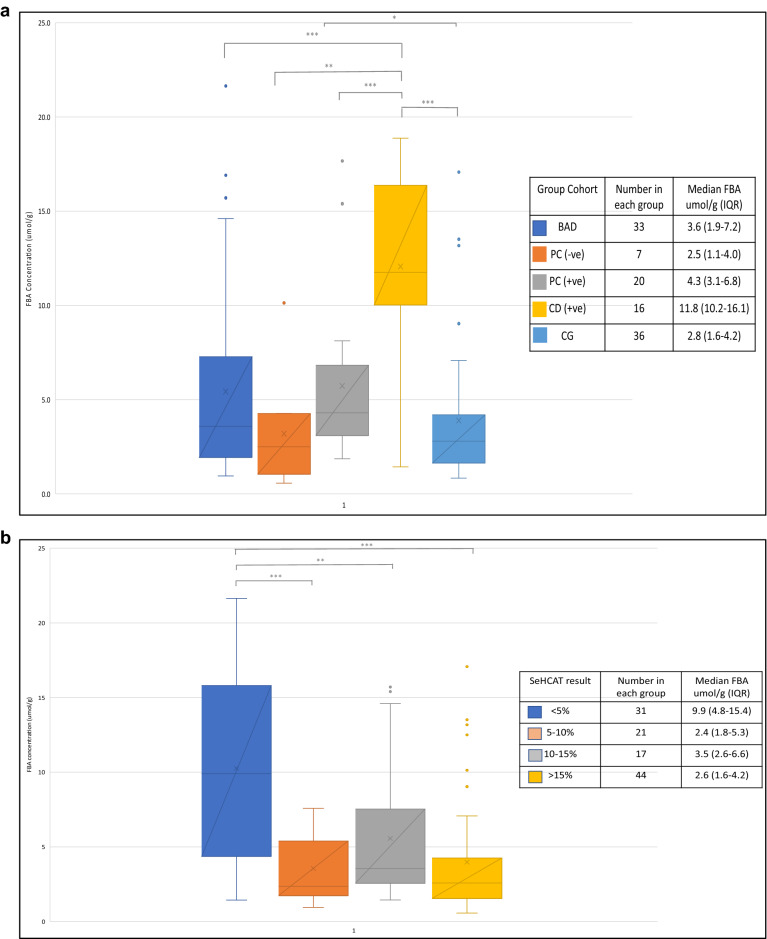
(**a**) FBA concentration in stool samples collected on enrolment to the study in the 4 different study groups—*FBA* faecal bile acids, *BAD* bile acid diarrhoea, *CD +ve* Crohn’s disease patients with a positive SeHCAT scan, *PC +ve* post-cholecystectomy patients with a positive SeHCAT scan, *PC* −*ve* post-cholecystectomy patients with a negative SeHCAT scan, *CG* SeHCAT-negative control group. The total number of patients and the median and interquartile range (IQR)s from each cohort are also listed. (**b**) Faecal bile acid (FBA) concentration in stool samples collected on enrolment to the study split into SeHCAT result where < 5% indicates severe BAD, 5–10% moderate, 10–15% mild and > 15% as normal result. The total number of patients and median and interquartile ranges (IQR) from each group are also listed. *p < 0.05, **p < 0.01 and ***p < 0.001.

In the SeHCAT positive patients, faecal bile acid concentrations and their IQR in patients in the idiopathic bile acid dairrhoea (3.6; 1.9–7.2 μmol/g), post-cholecystectomy (4.3; 3.1–6.8 μmol/g) and control group cohort (2.60; 1.6–4.1 μmol/g) were similar (p > 0.05; 1-β = 0.59–0.13) but all were significantly lower (p < 0.001) when compared to the Crohn’s disease cohort (11.8; 10.1–16.2 μmol/g) (Fig. 2a). In the SeHCAT negative patients, there was only one Crohn’s patient who had a faecal bile acid concentration of 12.5 μmol/g and there were 7 post-cholecystectomy patients with a median faecal bile acid concentration of 2.5 μmol/g (1.1–3.98). Table 1 provides the breakdown of median faecal bile acid concentrations per patient group based on their SeHCAT result.

**Table 1:** Total number (N) of patients and median FBA concentrations with IQR in each patient cohort based on their SeHCAT result. *BAD* idiopathic BAD, *PC* post-cholecystectomy, *CD* post-operative Crohn’s disease patients, *CG* SeHCAT negative control group.

SeHCAT result	BAD		PC		CD		CG	
	N	FBA	N	FBA	N	FBA	N	FBA
< 5%	7	8.2 (4.2–16.9)	8	5.6 (4.0–8.1)	16	11.8 (10.1–16.2)		
5–10%	12	2.1 (1.4–5.0)	9	3.2 (2.0–6.2)				
10–15%	14	3.2 (2.5–4.8)	3	2.8 (1.3–4.3)				
> 15%			7	2.5 (1.1–4.0)	1	12.5	36	2.8 (1.6–4.2)

**Figure 3:**
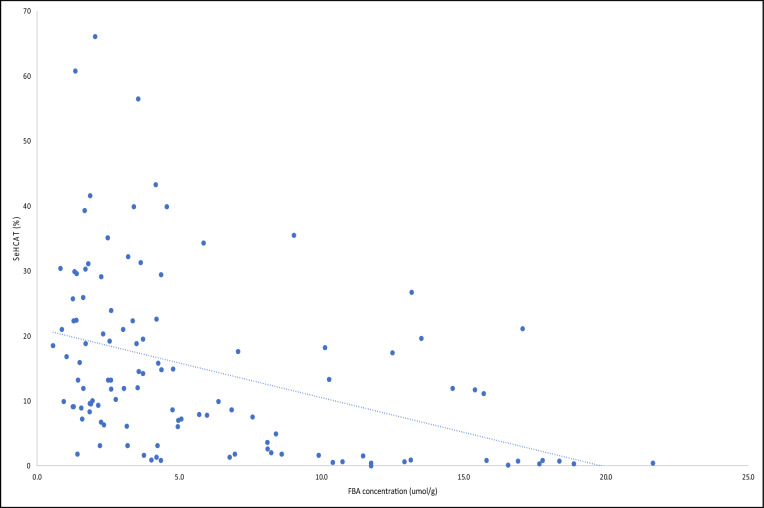
Spearman correlation of participants FBA concentration on enrolment to the study and SeHCAT retention time.

There was a significant difference in faecal bile acid concentrations and their IQR between patients who had a SeHCAT result < 15% (4.95; 2.6–10.5 μmol/g) and > 15% (2.6; 1.6–4.2 μmol/g), p < 0.001. Categorising faecal bile acid concentrations and their IQR in patients with severe, moderate, and mild disease showed significantly higher results (p < 0.001; 1-β = 0.55–0.12) with a SeHCAT retention of < 5% (9.9; 4.3–15.4 μmol/g) than in patients with SeHCAT retention of 5–10% (2.4; 1.8–5.3 μmol/g), 10–15% (3.5; 2.6–6.6 μmol/g) and > 15% (2.6; 1.6–4.2 μmol/g) (Fig. 2b). Faecal bile acid concentrations had a moderately negative correlation with SeHCAT % retention (Spearman r − 0.49; p < 0.0001; 1-β = 0.89–0.55) (Fig. 3).

### Faecal bile acid concentrations and clinical response to treatment

In total, 48 patients provided pre- and at least one post-treatment stool sample, either at visit 3 (4 weeks post-treatment) or visit 4 (8 weeks post-treatment). These patients were all given a trial of treatment with bile acid sequestrants (colesevelam at a starting dose of 625 mg once or twice daily depending on initial symptoms). Overall, the median faecal bile acid concentrations were not significantly different before (4.2; 2.7–9.2 μmol/g), at 4 weeks (4.8; 2.4–8.0 μmol/g), or at 8 weeks (4.8; 2.9–8.2 μmol/g) post-treatment (p > 0.05). After 8 weeks of treatment, 31/48 (64.6%) patients clinically responded to treatment, of which only 3 patients needed a dose escalation from twice daily to three times daily. From the 17 patients that did not clinically respond to treatment, dose escalation was attempted in 4 patients but did not result in an improvement of symptoms. Dose escalation was offered in the remaining 13 patients but was refused because they either experienced adverse effects with their initial dose, could not escalate due to concomitant medication or patient choice. No significant difference was demonstrated in faecal bile acid concentrations between pre-treatment, 4- or 8-weeks post treatment, in both responsive and non-responsive patients (p > 0.05). The median faecal bile acid concentrations in each of these groups are summarised in the Supplementary files-Table 2.

### Establishing sensitivity and specificity values

**Figure 4:**
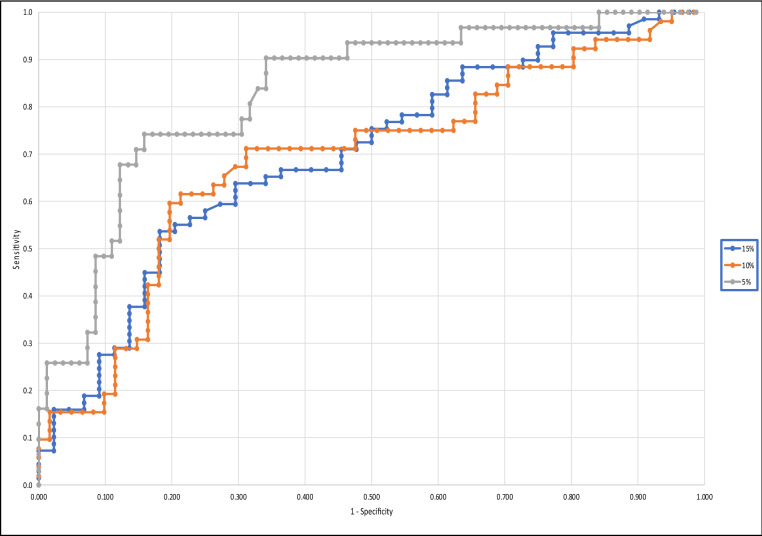
ROC curve demonstrating the clinical utility of faecal bile acid measurement for diagnosing severe bile acid diarrhoea (defined as SeHCAT retention of ≤ 5%), severe or moderate bile acid diarrhoea (defined as SeHCAT retention of ≤ 10%), and severe, moderate or mild bile acid diarrhoea (defined as SeHCAT retention of ≤ 15%).

As the diagnostic criteria to definitively exclude bile acid diarrhoea with a SeHCAT scan has not yet been universally agreed upon, we developed multiple ROC curves to explore diagnostic utility for faecal bile acid measurement (Fig. 4). When using a SeHCAT retention of ≤ 15% to confirm a diagnosis of bile acid diarrhoea, the area under the curve (AUC) was 0.698. At a faecal bile acid cut off of 1.6 μmol/g, sensitivity and specificity were 90% and 25%, respectively. This would result in 62 true positives and 11 true negative results. Whilst this would give only 7 false negatives, it would also result in 33 false positives. At a faecal bile acid cut off of 4.3 μmol/g, sensitivity and specificity were 57% and 77% respectively. At a faecal bile acid cut off of 10.1 μmol/g, sensitivity and specificity were 28% and 91%, respectively, giving 19 true positives and 40 true negative results. Whilst this cut off would result in 4 false positives, it would also result in 50 false negatives. Using a SeHCAT retention cut-off level of ≤ 10% and ≤ 5% to confirm bile acid diarrhoea gave an AUC of 0.689 and 0.832, respectively. This demonstrates that faecal bile acids have better diagnostic performance in diagnosing severe bile acid diarrhoea. However, the sensitivity and specificity percentages remain relatively poor with a high number of false positives or false negatives depending on whether a faecal bile acid cut off is chosen to optimise sensitivity or specificity.

## Discussion

A systematic review looking at clinical utility of tests for bile acid diarrhoea demonstrated an average reported sensitivity and specificity of SeHCAT to be 87.3% and 93.2%^8^. However, large variation is observed based on the cut off values for this test. A cut off of 8% gives a sensitivity and specificity of 67% and 97% respectively^8^, whilst a cut off value of 15% gives a sensitivity and specificity of 100% and 91% respectively^11^, and a cut off of 5% gives a result of 86% and 100% respectively^8^. Currently there are no clinical validity results for SeHCAT and the diagnostic accuracy has only been determined by treatment response with bile acid sequestrants^10^. As stated previously, clinical response to treatment is not an accurate measurement of bile acid diarrhoea as medication compliance and adherence is a serious concern for patients who cannot tolerate the medications^1,6,10^. Whilst the SeHCAT scan is the current gold standard for diagnosing bile acid diarrhoea in the UK, it has several disadvantages including radiation exposure, limited availability and time restraints for the patient who is required to come into hospital twice which may be difficult if they are working and/or are self-employed, have child-care responsibilities or have limited access to transportation. Jackson et al. further demonstrated that although a SeHCAT scan result can indicate the presence of malabsorption, it may not predict the variety or severity of patients’ symptoms as these are dependent on other factors, such as the amount of dietary fat intake^12^. Additionally, the SeHCAT test has limited accuracy at the indeterminate levels, with ill-defined normal ranges and thus likely variability between sampling. Therefore, there is a need for a test that is more easily accessible but still reliable and dependable in its results. In our study, we attempted to demonstrate efficacy with a convenient single, random stool sample that would be able to correctly identify bile acid diarrhoea and be the least disruptive to a patient’s lifestyle. Whilst our study demonstrated efficacy in patients with severe bile acid diarrhoea and/or in post-operative Crohn’s disease patients, there were several limitations to this test which we discuss below in greater detail.

In this study, faecal bile acid concentrations were significantly increased in post-terminal ileal resected Crohn’s patients with confirmed bile acid diarrhoea, compared to the other three groups. This is to be expected, given that in these patients, the primary location for bile acid reabsorption is removed. The results also demonstrated a significant difference in faecal bile acid results in patients with SeHCAT retention of < 5% compared to SeHCAT retention of > 15%, suggesting utility in diagnosing severe bile acid diarrhoea. There was, however, no significant difference in FBA concentrations in patients with idiopathic bile acid diarrhoea, post-cholecystectomy, or control group, suggesting that faecal bile acid measurement cannot reliably distinguish idiopathic bile acid diarrhoea or post-cholecystectomy patients from the control group cohort. On closer inspection, however, the post-operative Crohn’s cohort all had severe bile acid diarrhoea (< 5%) while 26/33 (78.8%) patients of idiopathic bile acid diarrhoea and 13/20 (65%) patients with post-cholecystectomy had a mixture of mild and moderate bile acid diarrhoea severity. The differences in severity grade between the groups may explain why there was such variability in the faecal bile acid concentrations and larger studies may help to expand on these findings.

The ROC curve analysis demonstrated that faecal bile acid analysis provided some utility in the diagnosis of bile acid diarrhoea, as the AUC was > 0.5, however no cut off provided an acceptable balance of sensitivity and specificity. It was therefore considered whether faecal bile acid measurement may be used to stratify patients requiring SeHCAT. A faecal bile acid concentration of 10.1 μmol/g demonstrated 91% specificity for severe, moderate or mild bile acid diarrhoea, therefore it may be that results above this value could be treated as bile acid diarrhoea, without requirement for SeHCAT referral. This may be of particular use in diagnosis of Type 1 bile acid diarrhoea in patients in whom there is a high diagnostic likelihood, such as those in our post-terminal ileal resected Crohn’s disease group. However, at this cut-off, in our cohort, there would have been 50/113 (44.2%) false negatives and therefore all patients with a result below 10.1 μmol/g would still require a SeHCAT referral; this would have been 90/113 (80%) patients in total, and therefore would not significantly reduce SeHCAT referral burden. The sensitivity and specificity achieved in this study using a faecal bile acid cut off of 1.6 μmol/g (using ≤ 15% SeHCAT retention as diagnostic of bile acid diarrhoea) were 90% and 25% respectively. At this cut off, there would have been 33 false positive results and 7 false negative results, out of 113 patients. As a result, 33 patients would have been unnecessarily treated with medication, which on top of everything else has its own cost implications.

The difficulty in achieving an acceptable balance of sensitivity and specificity may partly be due to the variability observed between individual stool samples, and as stated above, measuring faecal bile acids in a single random stool sample may result in a missed diagnosis. Camilleri et al. demonstrated that the faecal bile acid excretion in a random stool sample was closely correlated with the mean excretion in all samples from individual patients^13^. However, due to the variation in bile acid excretion per gram faecal weight in each bowel movement, and in the faecal pellet and supernatant of three of their patient samples, they concluded that individual stool samples were not representative of the total faecal bile acid excretion. Thus, more research may be required to determine the optimum stool sample for analysis, balancing both variation in excretion, and patient acceptability. A more recent paper suggested that a random stool sample could be used in conjunction with serum C4 for predicting bile acid diarrhoea^14^; however, this study used the random stool sample to assess percentage of primary bile acids, using a cut-off of > 10% primary bile acids in the sample, rather than total faecal bile acids. The Immunodiagnostik faecal bile acid kit being assessed in this study measures total faecal bile acids, not individual bile acids, and thus may not have the same utility.

The 48-h total faecal bile acid collection is the diagnostic method for bile acid diarrhoea in the United States where the ^75^SeHCAT scan is not licensed for use^15^. The diagnostic accuracy of this test has demonstrated an average sensitivity and specificity of 66.6% and 79.3% respectively. However, there are multiple problems with this method. The processes used to extract bile acids from faeces are complex and generally restricted to research laboratories. Furthermore, patients need to provide a 48-h stool sample and are required to undertake a 4-day strict 100 g fat-intake diet, making it inconvenient and unpleasant for the patient^16^. This requirement is due to the hypothesis that there is variable excretion in bile acids throughout the day in different stool samples, thus a random stool sample may result in a missed diagnosis^17^. The Immunodiagnostik assay uses a random stool sample, improving convenience for both patient and laboratory staff. However, use of a random sample may not provide a representative view of total faecal bile acid excretion, which may have contributed to the lack of significant results observed in the post-cholecystectomy and idiopathic bile acid diarrhoea groups, and also to the poor correlation observed with the SeHCAT scan results. In comparison, Sagar et al.^18^ found the median total faecal bile acids in bile acid diarrhoea to be higher than our results (9.17 (IQR 7.79–14.12) vs 4.8 (IQR 2.5–10.0) respectively). Whilst they used a random stool sample to analyse their results, their protocol differed from ours by using high performance liquid chromatography coupled to tandem mass spectrometry (HPLC–MS/MS). Their results also showed a greater concentration of secondary faecal bile acids compared to primary faecal bile acids (7.13 vs 1.5, respectively). The Immunodiagnostik assay is marketed as measuring total faecal bile acids, and consequently we were unable to separate primary and secondary faecal bile acids. Thus, our results are consistent with previous publications that a random stool sample may not provide sufficient information on faecal bile acid excretion^19,20^.

Measurement of faecal bile acids before and after treatment was performed to assess whether faecal bile acid measurement could assess therapeutic concordance and response. No significant difference was seen pre- and post-treatment, regardless of clinical improvement of symptoms. Camilleri et al.^19^ investigated the effect of colesevelam on faecal bile acids and found that post-treatment, there was an increase in deoxycholic acid with a reduction in cholic acid. There were not any differences, however, in the proportion of chenodeoxycholic acid, lithocolic acid or ursodeoxycholic acid. Their methodology also used a separate extraction technique using methanol which allowed them to extract bile acids that were both free and colesevelam-bound. As our assay did not have this critical methanol extraction step and only measures total faecal bile acids, it is likely that sequestered bile acids that are excreted are also measured in the assay, and therefore this method would not be appropriate for monitoring treatment efficacy.

Due to the global limited availability of the SeHCAT scan and the inconvenience of collecting a 48-h faecal bile acid sample, other methods to diagnose bile acid diarrhoea are currently being investigated, including the use of C4 and FGF-19 biomarkers. Vijayvargiya et al. recently demonstrated high negative predictive values and specificities for C4 (79%/83%) and FGF-19 (78%/78%) but found that combining the biomarkers, the sensitivity increased to 50% and the specificity was 65%^7^. Interestingly, their results improved when they excluded the cholecystectomy patients from their study. In our random stool samples, the post-cholecystectomy group did not show much variation in median faecal bile acid concentrations compared to the idiopathic bile acid diarrhoea cohort but it would be interesting to correlate these findings with C4 results.

The main strengths of our study include comparing our results against a control group and directly with the SeHCAT scan, the current gold standard diagnostic test for bile acid diarrhoea. As mentioned above, the main limitations of this study are the inability to differentiate between total and individual bile acids and from colesevelam-bound and free faecal bile acids with our assay. The use of a random stool sample may explain some of the negative results obtained, however this in itself is not considered a limitation, as the purpose of the study is to investigate whether the Immunodiagnostik kit could be used with a random stool sample for diagnosis of bile acid diarrhoea. Our study also did not control for other possible variables that could have affected our results, including dietary intake and definitively excluding other malabsorption conditions. Studies have shown that a high-fibre and low-fat diet results in lower faecal bile acid concentrations^21,22^. However, as patients undergoing SeHCAT scans are not advised to undergo any dietary restrictions before or during the testing time, we wanted to maintain the same guidance for consistency. As this was the first study to utilise this kit to diagnose bile acid diarrhoea, we appreciate that future larger studies exploring dietary modifications to changes in faecal bile acid concentrations would be beneficial. Faecal bile acid concentrations can also be elevated in the presence of small intestinal bacterial overgrowth (SIBO) and pancreatic insufficiency^23^. Whilst in an ideal world, all patients should have all of these conditions excluded, this is not practical in the real world and moreover, falls outside of the national BSG guidance on management of chronic diarrhoea^1^. Further investigations for chronic diarrhoea were left to the discretion of the patients’ standard care physician and was outside of the trial remit.

In this small study, we were able to demonstrate the potential efficacy of using faecal bile acids in diagnosing severe bile acid diarrhoea and/or in patients with post-operative Crohn’s disease, without the need for SeHCAT testing. This can prove to be beneficial in patients who do not have access to SeHCAT testing but are also unable to tolerate the bile acid sequestrant when given an empirical trial of treatment. Considering that patients with severe bile acid diarrhoea have a higher response rate to treatment, confirming their diagnosis via a single random faecal bile acid test could motivate them to persevere with their medication, with the goal to improve their underlying symptoms and quality of life. Whilst the correlation between the SeHCAT retention and faecal bile acid excretion is not strong enough to replace the utilisation of SeHCAT testing in our other patient groups, our calculations deduced that our study was not adequately powered. Thus, larger studies are now required to confirm the potential efficacy of using a single random faecal bile acid test to accurately diagnose bile acid diarrhoea in these patients in the absence of SeHCAT testing, establish a normal range, and determine whether a cut off can be set at which an acceptable sensitivity and specificity may be achieved.

## Supplementary Information

Supplementary Tables.

## Data Availability

Requests for any data, analytic methods and study materials will be considered and made available upon request to the corresponding author. Individual participant data will not be shared.
