# Supplementary material for: A single faecal bile acid stool test demonstrates potential efficacy in replacing SeHCAT testing for bile acid diarrhoea in selected patients
Source: Sci Rep. 2022 May 18;12:8313. doi: 10.1038/s41598-022-12003-z (PMC9117305; doi:10.1038/s41598-022-12003-z)
Supplement: Supplementary file 1 — Supplementary Tables. [file 41598_2022_12003_MOESM1_ESM.docx]

|  | | | **Total**  **(n=113)** | **BAD**  **(n=33)** | **CD**  **(n=17)** | **PC**  **(n=28)** | **CG**  **(n=35)** |
| --- | --- | --- | --- | --- | --- | --- | --- |
| **Age (years)** | | | 49.4 | 45.5 | 44.5 | 56.4 | 50.6 |
| **Gender** | | | | | | | |
|  | Male | | 44  (38.9%) | 18  (54.5%) | 7  (41.2%) | 10  (35.7%) | 9  (25.7%) |
|  | Female | | 68  (60.2%) | 15  (45.5%) | 10  (58.8%) | 17  (60.7%) | 26  (74.3%) |
|  | Unknown | | 1  (0.9%) | 0 | 0 | 1  (3.6%) | 0 |
| **Ethnicity** | | | | | | | |
|  | White British | | 94  (83.2%) | 24  (72.7%) | 14  (82.4%) | 24  (85.7%) | 32  (91.4%) |
|  | Indian Asian | | 6  (5.3%) | 5  (15.2%) | 0 | 1  (3.6%) | 0 |
|  | Black Caribbean | | 3  (2.7%) | 0 | 1  (5.6%) | 1  (3.6%) | 1  (2.9%) |
|  | Unknown | | 10  (8.8%) | 4  (12.1%) | 2  (11.8%) | 2  (7.1%) | 2  (5.7%) |
| **Co-morbidities** | | | | | | | |
|  | IHD | | 7  (6.2%) | 1  (3.0%) | 1  (5.6%) | 4  (14.3%) | 1  (2.9%) |
|  | Diabetes mellitus | | 6  (5.3%) | 2  (6.1%) | 0 | 2  (7.1%) | 2  (5.7%) |
|  | Dyspepsia/ Gastro-oesophageal reflux disease | | 10  (8.8%) | 3  (9.1%) | 0 | 4  (14.3%) | 3  (8.6%) |
|  | Malignancy | | 12  (10.6%) | 3  (9.1%) | 1  (5.6%) | 3  (10.7%) | 5  (14.2%) |
|  | Ulcerative colitis | | 4  (3.5%) | 3  (9.1%) | 0 | 1  (3.6%) | 0 |
|  | Crohn’s disease | | 19  (16.8%) | 2  (6.1%) | 17  (100%) | 1  (3.6%) | 0 |
|  | Anxiety/ Depression | | 14  (12.4%) | 3  (9.1%) | 0 | 2  (7.1%) | 9  (25.7%) |
|  | Chronic pain/ Fibromyalgia | | 8  (7.1%) | 1  (3.0%) | 0 | 2  (7.1%) | 5  (14.2%) |
|  | Others^a^ | | 43  (38.1%) | 10  (30.3%) | 5  (29.4%) | 13  (46.4%) | 15  (42.9%) |
|  | Nil | | 32  (28.3%) | 15  (45.5%) | 0 | 5  (17.9%) | 12  (34.3%) |
|  | Unknown | | 2  (1.8%) | 0 | 0 | 2  (7.1%) | 0 |
| **Medications** | | | | | | | |
|  | | Anti-depressants^b^ | 23  (20.4%) | 6  (18.2%) | 0 | 5  (17.9%) | 12  (34.3%) |
|  | | PPI | 32  (28.3%) | 12  (36.4%) | 0 | 14  (50%) | 6  (17.1%) |
|  | | Anti-spasmodics | 12  (10.6%) | 3  (9.1%) | 0 | 5  (17.9%) | 4  (11.4%) |
|  | | Analgesics^c^ | 18  (15.9%) | 3  (9.1%) | 1  (5.6%) | 5  (17.9%) | 9  (25.7%) |
|  | | Biologics | 5  (4.4%) | 2  (6.1%) | 2  (11.8%) | 0 | 1  (2.9%) |
|  | | Immunosuppressants | 6  (5.3%) | 1  (3.0%) | 5  (29.4%) | 0 | 0 |
|  | | Others^d^ | 43  (38.1%) | 10  (30.3%) | 4  (23.5%) | 14  (50%) | 15  (42.9%) |
|  | | Nil | 41  (36.3%) | 16  (48.5%) | 10  (58.8%) | 4  (14.3%) | 11  (31.4%) |
|  | | Unknown | 4  (3.5%) | 1  (3.0%) | 0 | 3  (10.7%) | 0 |

*Supplementary Table 1: Patient demographics in total and per group cohort. BAD: idiopathic bile acid diarrhoea, CD: post-operative terminal ileal resected Crohn’s disease, PC: post-cholecystectomy, CG: SeHCAT negative control group.*

1. *Includes hypertension, cerebrovascular disease, asthma or chronic obstructive pulmonary disease, hypercholesterolaemia, epilepsy, and hypothyroid disease.*
2. *Although generally prescribed for depression and/or anxiety, some patients were prescribed this for analgesic relief.*
3. *Includes co-codamol or other opioid derivative, non-steroidal anti-inflammatory agents, pregabalin or gabapentin.*
4. *Includes hypoglycaemics, anti-hypertensives, anti-epileptics, statins*

| **Clinical response** | | **Pre-treatment** | | **4-weeks**  **Post-treatment** | | **8-weeks**  **Post-treatment** | |
| --- | --- | --- | --- | --- | --- | --- | --- |
|  |  | **Faecal samples** | **Median FBA (IQR)** | **Faecal samples** | **Median FBA (IQR)** | **Faecal samples** | **Median FBA (IQR)** |
| **Total**  (n=48) | | 48 | 4.1  (2.0-9.2) | 46 | 4.8  (2.4-8.0) | 40 | 4.8  (2.9-8.2) |
| **Responders**  (n=31, 65%) | |  | 4.2  (2.7-10.2) | 30 | 4.8  (3.1-9.6) | 29 | 5.4  (2.8-9.5) |
| **Non-responders** (n=17, 35%) | |  | 2.6  (1.8-6.9) | 16 | 4.6  (2.2-7.2) | 11 | 4.2  (3.4-7.2) |
|  | | | | | | | |
| **SeHCAT < 5%**  (n=19) | | 19 | 9.9  (5.6-14.5) | 18 | 7.8  (6.3-17.5) | 16 | 9.1  (5.6-13.4) |
|  | **Responders**  (n=16, 84%) |  | 9.1  (4.4-12.1) | 16 | 7.8  (6.1-17.1) | 15 | 9.7  (5.9-15.1) |
|  | **Non-responders**  (n=3, 16%) |  | 15.8  (11.4-18.7) | 2 | 13.0  (9.8-16.3) | 2 | 6.9  (5.8-7.9) |
|  | | | | | | | |
| **SeHCAT 5-10%**  (n=15) | | 15 | 2.2  (1.6-4.0) | 15 | 3.4  (1.6-4.1) | 13 | 4.0  (2.8-5.4) |
|  | **Responders**  (n=9, 60%) |  | 2.2  (1.6-2.4) | 9 | 3.4  (1.6-4.0) | 9 | 4.0  (2.8-5.4) |
|  | **Non-responders**  (n=6, 40%) |  | 3.3  (1.6-6.0) | 6 | 3.1  (1.8-6.7) | 4 | 4.1  (3.4-5.0) |
|  | | | | | | | |
| **SeHCAT 10-15%**  (n=14) | | 14 | 2.7  (2.1-4.2) | 14 | 3.2  (2.3-5.8) | 11 | 3.4  (2.2-4.8) |
|  | **Responders**  (n=6, 43%)) |  | 3.3  (2.8-4.2) | 6 | 3.1  (2.1-4.2) | 6 | 2.8  (2.2-3.9) |
|  | **Non-responders**  (n=8, 57%) |  | 2.5  (1.8-3.1) | 8 | 4.0  (2.4-6.1) | 5 | 3.8  (3.3-5.6) |

*Supplementary Table 2: Median FBA concentrations and IQR at pre-treatment, 4- and 8-weeks post-treatment in patients who did and did not clinically respond to bile acid sequestrants based on their SeHCAT result. No significant difference was observed. Patients were excluded from post-treatment analysis if they failed to provide a faecal sample after treatment, if their SeHCAT result > 15% or if the clinical information was unavailable.*
